# Genomic and phenotypic characterization of *Salmonella enterica* serovar Kentucky

**DOI:** 10.1099/mgen.0.001089

**Published:** 2023-09-26

**Authors:** Amber K. Richards, Song Kue, Connor G. Norris, Nikki W. Shariat

**Affiliations:** ^1^​ Department of Population Health, University of Georgia, Athens, GA, USA; ^2^​ Center for Food Safety, University of Georgia, Griffin, GA, USA

**Keywords:** polyphyly, *Salmonella* Kentucky, WGS

## Abstract

Non-typhoidal *Salmonella* are extremely diverse and different serovars can exhibit varied phenotypes, including host adaptation and the ability to cause clinical illness in animals and humans. In the USA, *Salmonella enterica* serovar Kentucky is infrequently found to cause human illness, despite being the top serovar isolated from broiler chickens. Conversely, in Europe, this serovar falls in the top 10 serovars linked to human salmonellosis. Serovar Kentucky is polyphyletic and has two lineages, Kentucky-I and Kentucky-II; isolates belonging to Kentucky-I are frequently isolated from poultry in the USA, while Kentucky-II isolates tend to be associated with human illness. In this study, we analysed whole-genome sequences and associated metadata deposited in public databases between 2017 and 2021 by federal agencies to determine serovar Kentucky incidence across different animal and human sources. Of 5151 genomes, 90.3 % were from isolates that came from broilers, while 5.9 % were from humans and 3.0 % were from cattle. Kentucky-I isolates were associated with broilers, while isolates belonging to Kentucky-II and a new lineage, Kentucky-III, were more commonly associated with cattle and humans. Very few serovar Kentucky isolates were associated with turkey and swine sources. Phylogenetic analyses showed that Kentucky-III genomes were more closely related to Kentucky-I, and this was confirmed by CRISPR-typing and multilocus sequence typing (MLST). In a macrophage assay, serovar Kentucky-II isolates were able to replicate over eight times better than Kentucky-I isolates. Analysis of virulence factors showed unique patterns across these three groups, and these differences may be linked to their association with different hosts.

## Data Summary

The whole-genome sequences analysed in this study are openly available from the National Center for Biotechnology Information (NCBI) database; the short-read archive accession numbers are listed in Table S1 (available with the online version of this article).

**Impact Statement**

*
Salmonella enterica* serovar Kentucky is the most isolated serovar from broiler chickens in the USA. Although this serovar is rarely associated with human illnesses domestically, it is frequently reported in human clinical cases worldwide. Serovar Kentucky is polyphyletic with two distinct lineages, Kentucky-I and -II, that are more related to other serovars than themselves. Phylogenetic analysis of serovar Kentucky whole-genome sequences revealed the presence of another distinct lineage (Kentucky-III), which is more closely related to Kentucky-I than Kentucky-II. Assessment of 5151 genomes from isolates collected from food animals and humans from 2017 to 2021 showed that serovar Kentucky-I isolates are most commonly found in broilers, while Kentucky-II and -III are typically isolated from cattle and humans. Further analysis showed that the three groups exhibited different suites of virulence factors and that Kentucky-II isolates replicated in macrophages better than Kentucky-I isolates. This work highlights the need for *Salmonella* characterization beyond serovar identity.

## Introduction

Non-typhoidal *Salmonella* is one of the most frequently isolated bacterial food-borne pathogens in the world and is considered a significant threat to public health [1, 2]. There are over 2600 distinct serovars that belong to *Salmonella enterica* that are each defined by a unique combination of 46 different somatic (O) and 119 flagella (H) antigens according to the Kaufman–White–Le Minor (KWL) scheme [3]. Over half of the serovars belong to *S. enterica* subspecies *enterica *, with approximately 50 serovars accounting for 99 % of all isolates found from humans and domestic food animals [4]. The KWL scheme can be used to phenotypically serotype the expressed O and H antigens on the surfaces of each *Salmonella* by agglutination. *Salmonella* serovars can also be predicted from whole-genome sequence (WGS) data that includes analysis of the genes that encode the O and H antigens [5, 6].

Different *Salmonella* serovars can display an impressive repertoire of genetic and phenotypic heterogeneity that can manifest by their abilities to colonize different hosts and/or express different virulence genes [7–10]. Adding to this complexity, an estimated 10 % of *S. enterica* subsp. *enterica* serovars are polyphyletic and consist of two or more distinct genetic lineages that do not share a recent common ancestor [11]. Serovars Newport and Montevideo are two examples of polyphyletic serovars. Serovar Newport has three lineages, groups I–III, and specific host association has been demonstrated for some of these lineages [12–15]. Similarly, serovar Montevideo has four different lineages, I–IV, that have been attributed to different sources [16]. Therefore, despite sharing their O and H antigens, polyphyletic members of a serovar can be quite distinct and these differences are not resolved by their antigenic KWL schemata alone. Additional subtyping approaches are required to distinguish between polyphyletic isolates of the same serovar [11, 17–21].

Serovar Kentucky is polyphyletic, and this is reflected by different genetic analyses, including PFGE, CRISPR-typing, multilocus sequence typing (MLST) and whole-genome sequencing [22–26]. Vosik *et al*. [23] designated the two serovar Kentucky lineages as group I (Kentucky-I) and group II (Kentucky-II), following the established nomenclature for serovar Newport [12]. Using MLST, the two groups fall into two major different sequence types (STs), ST152 and ST198 [24]. The Kentucky-I isolates belong to ST152 and Kentucky-II to ST198 [23]. Serovar Kentucky-I isolates have been found in poultry and cattle and their associated environments [24, 27]. For many years, it has been the number one serovar isolated from broiler chickens at processing [28, 29] and studies have shown that these are typically isolates belonging to the Kentucky-I lineage [24, 27]. Despite broilers being the largest single reservoir for human cases of *Salmonella* [30], serovar Kentucky is rarely associated with human clinical cases in the USA [31]. This suggests that humans come into contact frequently with serovar Kentucky-I but that this contact does not result in significant illness. Conversely, serovar Kentucky-II has a higher association with human illness outside of the USA, particularly in Europe, South-East Asia, China and Africa [25, 32–34]. In Europe, serovar Kentucky is the seventh most common serovar to cause human illness and some ST198 cases have been linked to travel in Africa [35, 36]. Some cases of salmonellosis caused by serovar Kentucky-II isolates in the USA have been linked to international travel [37].

Based on the potential higher association of serovar Kentucky-II isolates with human illness, it is important to monitor the prevalence of isolates belonging to this lineage in food animal production. Because serovar Kentucky-I and -II isolates cannot be distinguished by traditional serotyping or by only considering predicted serovars from SeqSero [6] or sistr [5], higher-resolution approaches are required to monitor this serovar [38]. In this study, we sought to survey serovar Kentucky incidence in the USA from 2017 to 2021 across food animals and humans, and to discern genomic and pathogenic differences across this serovar.

## Methods

### Genomes and genome assemblies

A total of 414 short-read WGSs computed as *S *. *enterica* serovar Kentucky were downloaded on March 16 2022 from Pathogen Detection via the National Center for Biotechnology Information (NCBI) using fastq-dump [39]. These genomes were isolated from broiler, swine, bovine, turkey and human sources representing each SNP cluster (260 genomes) as well as isolates with no designated SNP cluster (154 genomes), which we refer to as singletons. Genomes were selected based on their SNP cluster, since isolates within the same cluster are considered similar (within 50 nucleotides). Although the SNP cluster attribution changes as more genomes are added to Pathogen Detection, we chose to select genomes using this information as it allowed us to impartially use the minimal number of genomes while still encompassing the entire genomic diversity of this serovar. The ‘computed types’ filter, which uses the SeqSero serovar prediction, was used to identify genomes belonging to serovar Kentucky to prevent analysis of genomes with incorrectly attributed serovars [6]. The Pathogen Detection output was further filtered to analyse isolates uploaded by the United States Department of Agriculture–Food Safety and Inspection Service (USDA-FSIS; broiler, turkey, swine and bovine sources) and the Centers for Disease Control and Prevention (CDC; human sources). Since data from the former represents *Salmonella* from routine surveillance in food animal production, this avoids potential over or under representation of some genetic lineages that might occur with other sample types. The metadata associated with each genome was used for source attribution. Additionally, the metadata from all *S. enterica* subspecies *enterica* isolates uploaded by the USDA-FSIS (broiler, turkey, swine and bovine sources) and the CDC (human sources) between 1/1/2017 and 31/12/2021 was downloaded as a csv file on March 26 2022, from Pathogen Detection. The computed types filter identified the serovar and Excel was used to determine the serovar that occurred most frequently in each source.

The list of 414 genomes taken from Pathogen Detection plus the 2 isolates in our collection are presented in Table S1, along with serovars Agona (ATCC 51957) and Worthington (ATCC 9607), which were used as phylogenetic outgroups. Genomes were *de novo* assembled using SPAdes (v3.15.4), BBMap (v38.98) was used to remove contigs less than 500 nucleotides from the fasta files [40], and quast (v5.0.2) was used to determine the quality of the assemblies [41–43]. Genomes with less than 150 contigs were selected for phylogenetic analysis, with the exception of genomes with lower assembly statistics that were the only representative isolate of a particular SNP cluster (and/or animal/human source within a cluster) or that were singletons (i.e. not attributed to a SNP cluster).

### Phylogenetic analyses

For all phylogenetic analyses, kSNP (v4.0) was used to generate a core-SNP data matrix using *k*-mer size 17 and *S. enterica* subspecies Typhimurium LT2 as the annotated genome [44]. RAxML (v8.2.12) was used to perform auto Majority Rule Criterion (autoMRE) under the GTRCAT model for the rapid bootstrapping. The GTRGAMMA model was used to infer the final best scoring maximum-likelihood tree [45]. iTOL (v6) was used to annotate and visualize phylogenetic trees [46]. To trim the number of Kentucky-I and Kentucky-II isolates to a reasonable number to generate a phylogeny of the entire serovar, we used Phylogenetic Diversity Analyzer [47] on the individual trees that were generated for Kentucky-I genomes and for the Kentucky-II genomes to select 10 of each lineage.

### Subtyping analyses and virulence factor identification

Isolates curated from Pathogen Detection were typed using the Achtman 7 gene MLST scheme in EnteroBase [48, 49]. Isolates 8E00076 and M11023374001A were typed using the web-based application pubMLST [50]. CRISPRViz was used to identify the CRISPR spacers from the assembled genomes [51] and the CRISPR spacers were visualized using an Excel-based macro as described [52]. CRISPR type was assigned as previously described, based on the combination of CRISPR1 and CRISPR2 alleles in each genome [53]. The CRISPR types of seven singletons could not be resolved due to poor assemblies. Putative virulence factors were identified by downloading the Virulence Factor Database (VFDB) and searching for *Salmonella* virulence factors against the genomes using blast+ locally with a minimum threshold of 75 % nucleotide identity and 75 % query coverage, and the top hit was selected [54]. Hits with the highest per cent identity of each virulence factor against VFDB were then curated into a heatmap.

### Bacterial isolates and whole-genome sequencing

Two previously characterized human clinical isolates of *S *. *enterica* serovar Kentucky (8E00076 and M11023374001A) were used in this study [23], and the two poultry isolates (FSIS11812464 and FSIS11813932) were obtained from the USDA-FSIS. Single colonies of each strain were grown overnight in LB broth at 37 °C and then stored in glycerol at −80 °C. For the two human isolates, genomic DNA was isolated from the overnight culture using the Promega Genome Wizard kit. Sequencing was performed at Wright Labs on an Illumina NextSeq system (150 cycle, paired end).

### Quantitative PCR (qPCR) screening for serovar Kentucky-II isolates in serovar Kentucky isolated from poultry

A total of 1642 serovar Kentucky isolates collected by the Georgia Poultry Laboratory Network from January 2020 to December 2021 were shared with us. Colony picks were used to screen for Kentucky-I or Kentucky-II using the following TaqMan-based qPCR assays (all sequences are in the 5´−3´ orientation): Kentucky-I forward primer, gataaaccgcgcccctcac; reverse primer, cgcggggaacacaaatcgtt; probe, FAM-ccgcgttaaacatagttgccggtttatcccc; Kentucky-II forward primer, taaaccgccgccttcacc; reverse primer, ggggaacacgttgcgg; probe, FAM-cggtgcgcgtcagccagc. IDT PRIME master mix was used, and the cycling parameters were as follows: 95 °C for 3 min, followed by 40 cycles of 15 s at 95 °C and 30 s at 55 °C. The qPCR assays were performed on the qTower3 platform and analysed using qPCRsoft 4.0 software (Analytik Jena). For each qPCR, a positive control for each lineage was included, along with a negative water-only control.

### Macrophage assay

Murine macrophage cells, RAW264.7, were maintained in a T-75 cell culture flask in Dulbecco’s modified Eagle's medium (DMEM) with 10 % (v/v) FBS and 1 % (v/v) penicillin/streptomycin at 37 °C with 5 % CO_2_. Cells were harvested or propagated once they reached a confluency of 60–75 %. The macrophage assay quantified *Salmonella* association, invasion and replication, following a published protocol [55]. The protocol was modified to perform the assay in 24-well plates by seeding macrophages at a concentration of 7.5×10^4^ cells ml^−1^ and inoculating with 1.5×10^7^ c.f.u. *Salmonella *. Each *Salmonella* isolate was inoculated in technical triplicate and the experiment was performed independently three times. Statistical significance between *Salmonella* isolates at each stage (association, invasion, replication) was determined by a Tukey HSD test using sas (v3.8).

## Results

### Overview of *S *. *enterica* serovar Kentucky genomic structure

**Fig. 1.**
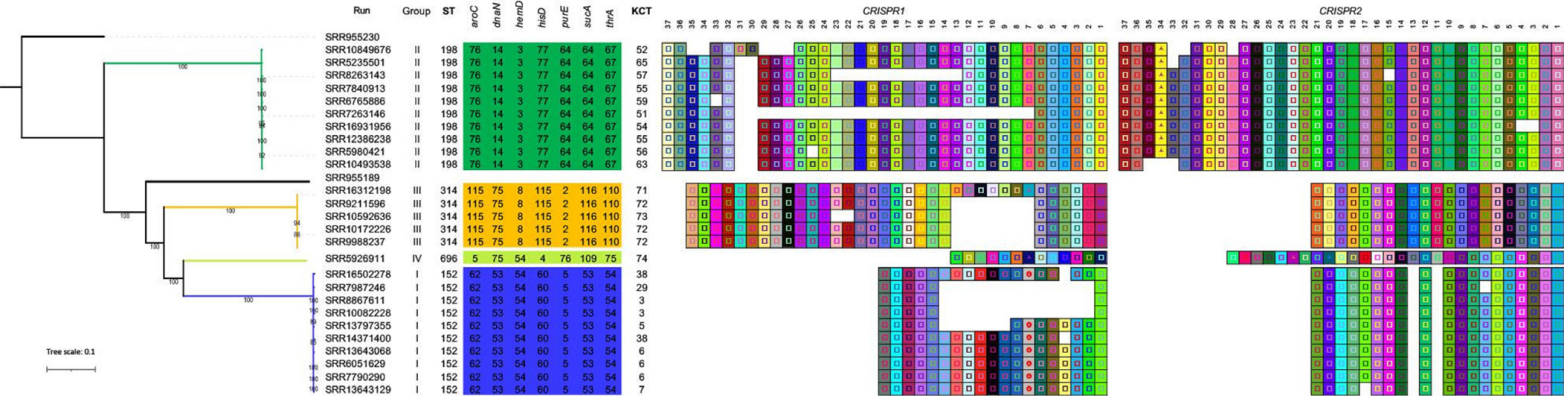
*
S *. *enterica* serovar Kentucky is separated into four polyphyletic lineages. kSNP was used to generate a SNP matrix for 26 serovar Kentucky genomes and 2 outgroup genomes [serovar Worthington ATCC 9607 (SRR955230) and Agona ATCC 51957 (SRR955189)]. The phylogenetic tree was generated using RAxML and visualized using iTOL; bootstrap values greater than or equal to 70 are shown. The scale bar represents the number of nucleotide substitutions per site. Serovar Kentucky-I genomes are indicated by blue branches, Kentucky-II by green branches, and the new lineages, Kentucky-III and Kentucky-IV, by yellow and light green branches, respectively. The MLST allelic profiles and CRISPR serovar Kentucky Sequence Types (KSTs) are also shown. For the two CRISPR arrays, the direct repeats are omitted for clarity and each uniquely coloured box represents a unique spacer sequence.

A total of 5151 *S *. *enterica* serovar Kentucky genomes were obtained from the Pathogen Detection platform on NCBI. All isolates were uploaded between January 2017 and December 2021 and collected by either the USDA-FSIS or CDC. In total, 418 *Salmonella* genomes were analysed, which included 414 serovar Kentucky genomes from Pathogen Detection that represented 185 unique SNP clusters (260 genomes) and also 154 singleton genomes. Two human clinical isolates, 8E00076 and M11023374001A, were sequenced and included in the phylogenetic analysis and genomes from serovar Agona (ATCC 51957) and serovar Worthington (ATCC 9607) were used as outgroups. Table S1 contains a full list of genomes investigated in this study. The mean genome size for the group of 411 genomes was 4.91 Mb, and the mean number of contigs and N50 were 76.7 and 205.91, respectively (Table 1). A cladeogram shows the separation of Kentucky-I and -II and suggests that there are two other lineages (yellow and black clades in Fig. S1) that are more closely related to group I than group II. To investigate the overall phylogenetic structure of serovar Kentucky, a tree was built using ten genomes each from groups I and II and six additional genomes belonging to the two new clades (Fig. 1). Our phylogenetic analyses confirmed the polyphyletic relationship between the Kentucky-I (blue branches) and Kentucky-II (green) lineages, which were separated by serovar Agona. Two other distinct clades were observed, and we termed these group III (Kentucky-III; orange) and group IV (Kentucky-IV; light green). There was no overlap of the MLST or CRISPR types between isolates belonging to the four lineages. There was a single allele (*dnaN*) shared between group III and group IV genomes and a few CRISPR2 spacers shared between groups III and IV and between groups IV and I. These were all older spacers (with respect to spacer acquisition). There were no shared spacers in the CRISPR1 arrays between any of the three groups. This data demonstrates that serovar Kentucky is represented by four polyphyletic lineages.

**Table 1.** Genome assembly statistics

Characteristic	Mean value	Standard deviation	Minimum	Maximum
Genome size (Mb)	4.91	0.095	4.67	5.14
No. of contigs	76.7	34.46	12	271
N50 value (kb)	205.91	127.09	34.69	774.12

**Fig. 2.**
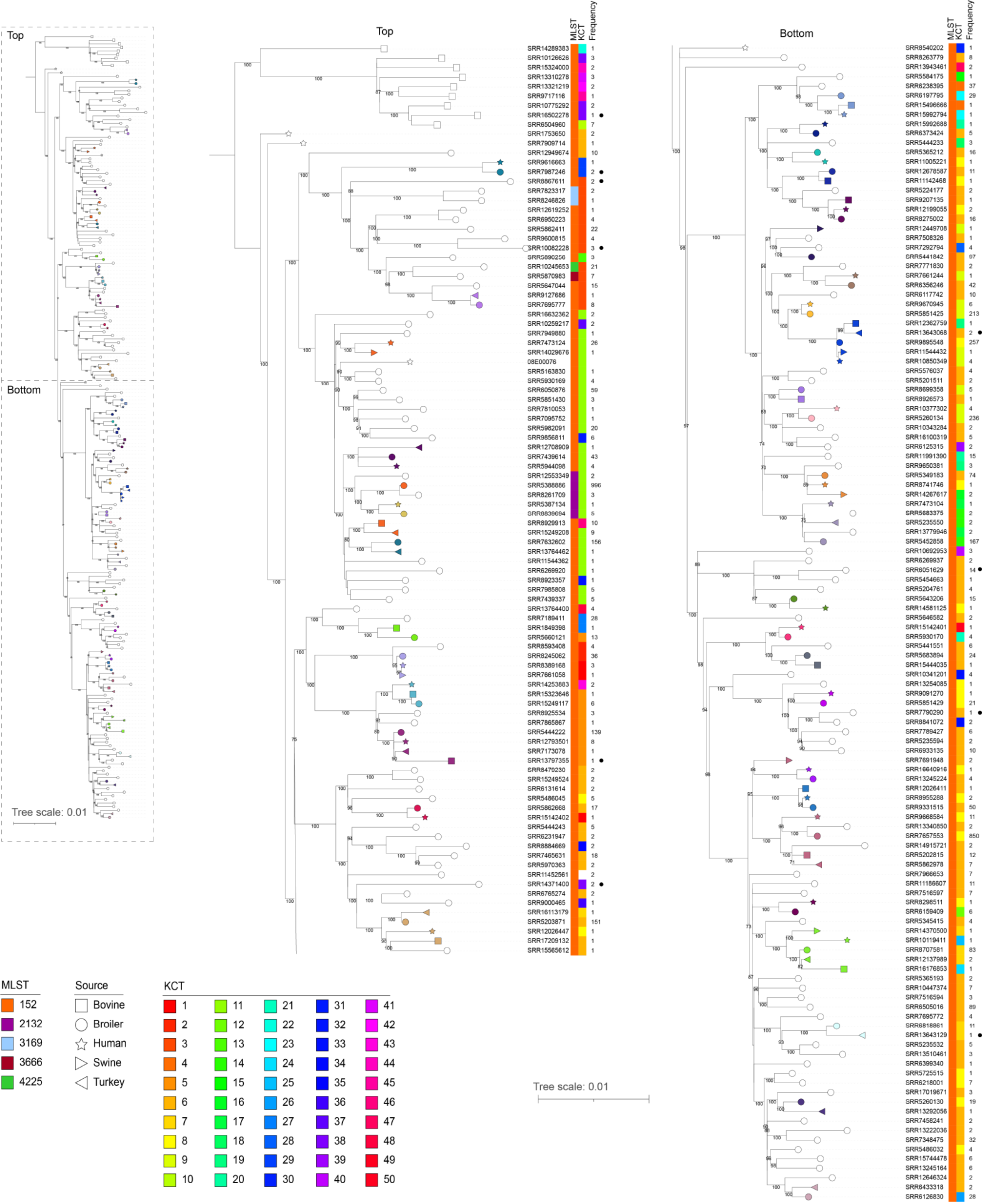
*
S. enterica* Kentucky-I phylogeny. kSNP was used to generate a SNP matrix for 218 serovar Kentucky-I genomes. The phylogenetic tree was generated using RAxML and visualized using iTOL; bootstrap values greater than or equal to 70 are shown. The scale bar represents the number of nucleotide substitutions per site. Shapes on the branch tips represent the source, as indicated in the key. White shapes represent a SNP cluster that only contained isolates from that source. Coloured shapes represent multi-source SNP clusters and are colour-coded according to the matching SNP cluster. The MLST types and KCTs are shown to the right of the tree as indicated. The black dots represent the isolates selected for inclusion in Fig. 1.

**Fig. 3.**
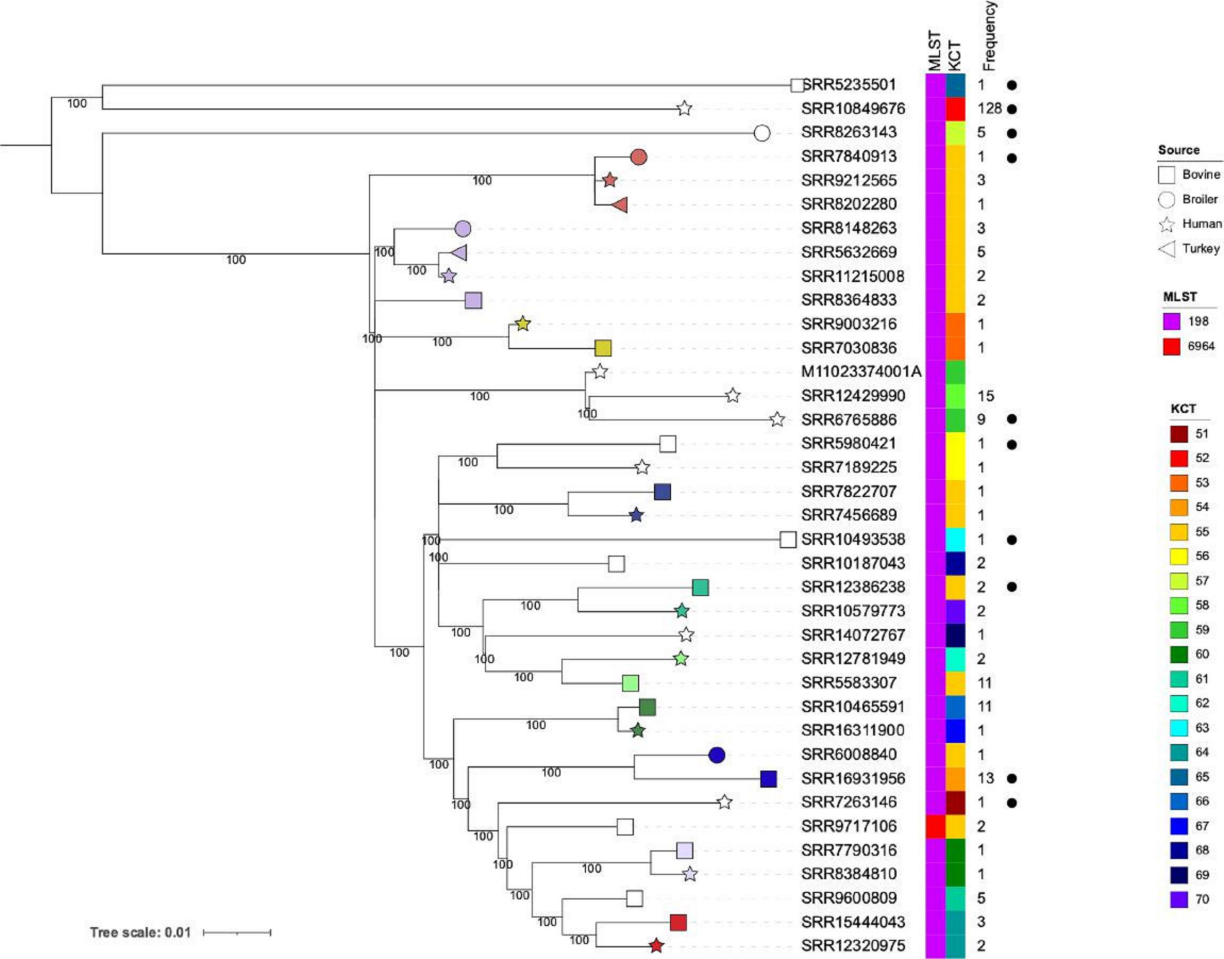
*
S *. *enterica* Kentucky-II phylogeny. kSNP was used to generate a SNP matrix for 37 serovar Kentucky-I genomes. The phylogenetic tree was generated using RAxML and visualized using iTOL; bootstrap values greater than or equal to 70 are shown. The scale bar represents the number of nucleotide substitutions per site. Shapes on the branch tips represent the source, as indicated in the key. White shapes represent a SNP cluster that only contained isolates from that source. Coloured shapes represent multi-source SNP clusters and are colour-coded according to matching SNP cluster. The MLST types and KCTs are shown to the right of the tree as indicated in the key. The black dots represent the isolates selected for inclusion in Fig. 1.

Given the large number of Kentucky-I and -II genomes, we analysed the phylogeny of these two groups. Given the genetic disparity between these two lineages, we performed the analysis on them separately so as to provide a larger core genome and, therefore, provide a higher-resolution analysis. Phylogenetic trees for Kentucky-I and -II showing just genomes attributed to a SNP cluster, plus the human isolates sequenced here, are shown in Figs 2 and 3, respectively. One representative genome from one of each of the five sources was included per SNP cluster for a total of 148 broiler, 39 bovine, 13 swine, 51 human and 9 turkey genomes. Within Kentucky-I, there were 156 SNP clusters represented, and cluster PDS000097081.50 contained the largest number of isolates (1042) (Fig. 2, Table S1). There were four SNP clusters that contained serovar Kentucky genomes from all five sources (PDS000029448.246, PDS000032514.79, PDS000097081.50 and PDS000097397.43), and all were Kentucky-I. Within the Kentucky-I tree, there was a distinct clade that consisted of nine genomes solely from cattle isolates (top of top panel, squares, Fig. 2). There were two evolutionarily distinct genomes from human isolates (SRR1753650 and SRR7909714) without closely related isolates from an animal source. Otherwise, across the entire Kentucky-I phylogeny, broilers were a source of all but one SNP cluster (SRR8540202; PDS000039066.7). Among the Kentucky-II genomes, there were 23 unique SNP clusters, and cattle isolates were represented throughout the tree (Fig. 3). There were three genomes that clustered together; these represent 25 isolates that are all from human isolates, and there were no closely related isolates from animal sources.

### Subtyping analysis

Among all serovar Kentucky genomes, there were 15 distinct MLST types represented (the genome for PNUSAS122906 was missing the *thrA* gene) (Table S1). For Kentucky-I, 93.8 % (331/352) of the genomes belonged to ST152 and consisted of 230 broiler isolates, 44 bovine isolates, 35 human isolates, 15 swine isolates and 7 turkey isolates. There were 22 serovar Kentucky-I isolates that belonged to additional STs including, ST2132 (*n*=8), ST3169 (*n*=5) and ST4225 (*n*=2). Collectively, across Kentucky-I genomes, there were 75 distinct serovar Kentucky CRISPR types (KCTs), KCT1–KCT50, KCT75–KCT90, KCT100–KCT107 and KCT109. All 75 KCTs were found within isolates that typed as ST152. Seven of the eight ST2132 genomes typed as KCT11 with the remaining genome belonging to KCT37. All genomes belonging to ST3169, ST3666 and ST4225 belonged to KCT3. ST4012 and ST4580 genomes were both typed as KCT6. There were two allelic differences between ST3169 and ST3666 and ST4225 (*purE* and *sucA*), and one allelic difference between ST3666 and ST4225 (*sucA*). For serovar Kentucky-II isolates, 52 belonged to ST198 and 1 isolate belonged to ST6964 (Table S1). ST198 was represented by 29 different KCTs (KCT51–KCT70, KCT91–KCT-98, KCT108) and the ST6964 isolate (SRR9717106) typed as KCT55. ST198 and ST6964 differ by the *purE* allele. Seven serovar Kentucky-III isolates belonged to ST314 and these were represented by three KCTs, KCT71–KCT73. One serovar Kentucky-III isolate (PNUSAS122906) did not have a designated ST due to missing the *thrA* allele. This isolate was represented by KCT99, and was the only isolate with this KCT. The Kentucky-IV isolates (SRR5926911 and SRR10615607) typed as ST696 by MLST and KCT74 by CRISPR-typing, both of which were unique to this isolate. Overall, CRISPR-typing identified 109 unique KCTs, with KCT6 and KCT55 being the most frequently identified in serovar Kentucky- I and -II, respectively (Figs 2, 3 and S2).

### Distribution of serovar Kentucky across different food animals

**Fig. 4.**
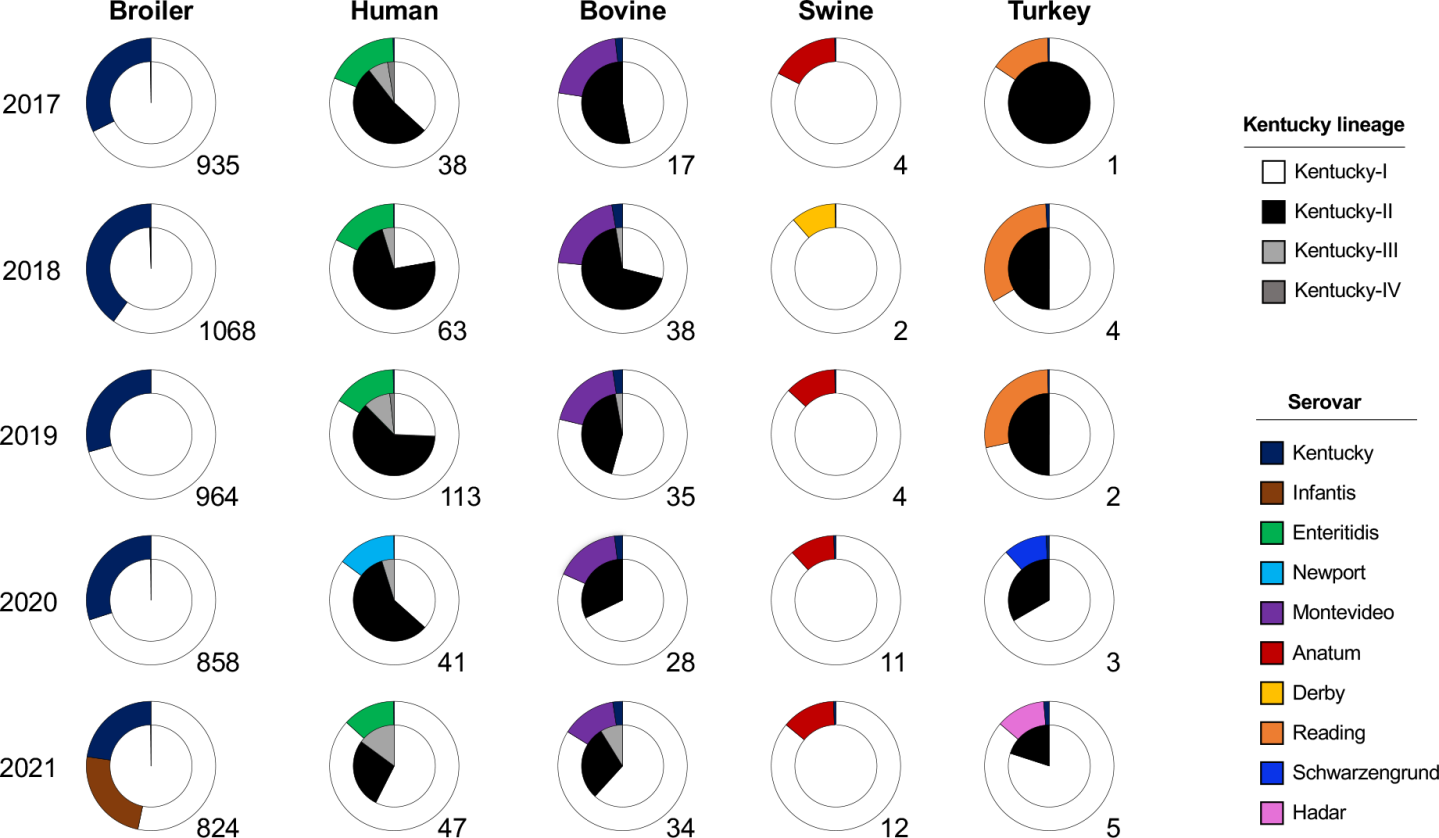
Distribution of *S *. *enterica* serovar Kentucky in USA food animal production systems and human clinical cases, 2017–2021. Source information was acquired from metadata associated with each WGS uploaded to the NCBI Pathogen Detection database. The inner white pie slices represent Kentucky-I; black, Kentucky-II; and grey, Kentucky-III. The two outlier isolates that did not belong to these three groups (ST696) are shown in dark grey and only occurred in humans (2017 and 2019). The number beside each pie chart denotes the total number of serovar Kentucky isolates from the respective source identified in that year. The outer circle represents the proportion of the most prevalent serovar compared to serovar Kentucky in each animal reservoir as determined by the metadata available on Pathogen Detection.

Since food animals are a significant source of human salmonellosis, we next sought to identify how the three groups of serovar Kentucky were distributed across different food animals and humans over a 5 year period from 2017 to 2021. Overall, the incidence of serovar Kentucky in food animals was highest in broilers, accounting for 23.09 %(4649/20 133) of *S *. *enterica* Kentucky isolates defined by the metadata on Pathogen Detection and according to our search parameters (Fig. 4, Table 2). Concordantly, serovar Kentucky was the major serovar in broilers from 2017 to 2020 but not in any other animal source, nor humans (Fig. 4). Serovar Kentucky was surpassed by serovar Infantis in 2021 as the most frequent serovar in broilers at processing. The lowest prevalence of serovar Kentucky was in turkeys (0.78 %; 15/1935) (Table 2), where serovars Reading, Schwarzengrund and Hadar were typically the most prevalent (Fig. 4). In broilers, serovar Kentucky-I was most prevalent (99.8 %, 4638/4649; white portion of inner circle in Fig. 4) compared to Kentucky-II (0.2 %; black portion of inner circle). This agrees with qPCR assays we performed to screen 1642 chicken serovar Kentucky isolates collected by the Georgia Poultry Laboratory Network from January 2020 to December 2021, where we identified just a single serovar Kentucky-II isolate. In swine, 100 % (33/33) of isolates belonged to Kentucky-I. In terms of animal reservoirs, Kentucky-II isolates were most often found in cattle and in 2018 they accounted for 68 % (26/38) of all serovar Kentucky isolated from bovine sources. Kentucky-II isolates were also identified in turkeys; however, the overall incidence of serovar Kentucky was very low in turkeys compared to other food animals. The greatest relative proportion of Kentucky-II was in humans, with an overall incidence of 57.3 % (173/302). Interestingly, following a peak in 2018 where 73 % of serovar Kentucky isolates were group II, the relative number of isolates belonging to this lineage decreased each year to 28 % in 2021. Concurrently, the relative proportion of Kentucky-III (grey slices) isolates from humans has increased since 2018, as well as overall in bovine isolates. Serovar Kentucky isolates belonging to this lineage were not detected in the other animal sources.

**Table 2.** Incidence of *S *. *enterica* serovar Kentucky by source between 2017 and 2021

Year/incidence	* Salmonella *	Source				
		Broiler	Human	Bovine	Swine	Turkey
2017	Kentucky	935	38	17	4	1
	All *Salmonella *	3825	12 602	922	1356	244
2018	Kentucky	1068	63	38	2	4
	All *Salmonella *	4716	31 374	1421	1296	466
2019	Kentucky	964	113	35	4	2
	All *Salmonella *	4242	41 252	1440	1332	491
2020	Kentucky	858	41	28	11	3
	All *Salmonella *	3721	40 358	1384	1990	377
2021	Kentucky	824	47	34	12	5
	All *Salmonella *	3629	30 082	1400	1963	357
Total	Kentucky	4649	302	152	33	15
	All *Salmonella *	20 133	1 55 668	6567	7937	1935
Kentucky incidence (%)		23.09	0.19	2.31	0.42	0.78

### Macrophage assay to assess serovar Kentucky virulence

**Fig. 5.**
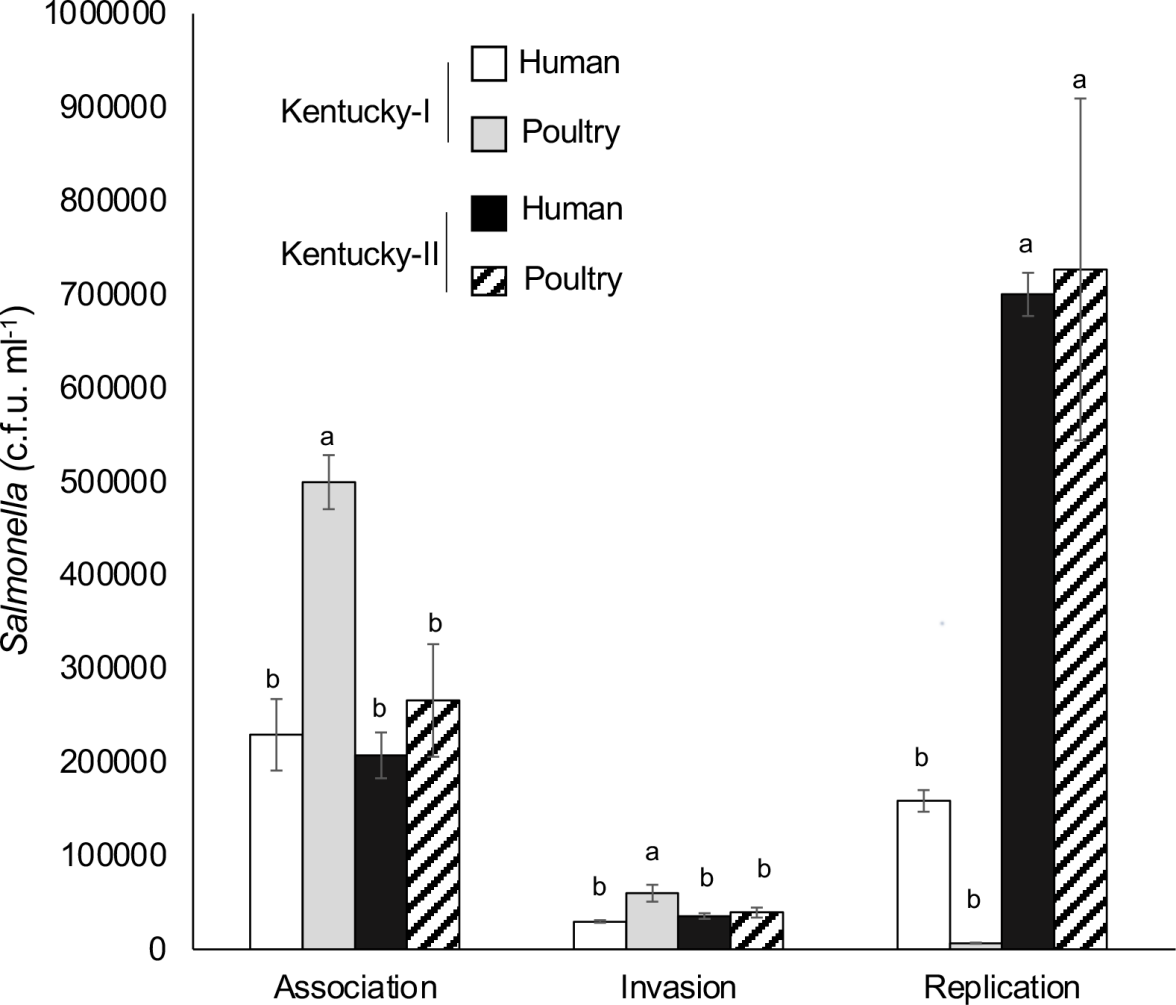
*
S *. *enterica* serovar Kentucky-II isolates replicate better than Kentucky-I isolates in macrophages. Four *S *. *enterica* serovar Kentucky isolates were used to infect RAW264.7 murine macrophage cells and *Salmonella* was quantified to determine differences in attachment, invasion and replication among the isolates. Error bars represent the standard error of the three technical replicates. The Tukey–Kramer HSD test was used, and significance represented by lowercase letters. Different letters represent significant difference between isolates independently for each of the three assays.

Given that serovar Kentucky-II isolates are more commonly found in humans compared to Kentucky-I isolates and that the proportion of human illnesses caused by serovar Kentucky is greater outside of the USA where Kentucky-II isolates are more often observed, we performed a macrophage assay to assess virulence differences among different serovar Kentucky isolates. Two serovar Kentucky-I isolates, one from poultry (FSIS11812464) and one from a human (8E00076), and two Kentucky-II isolates, one from poultry (FSIS11813932) and one from a human (M11023374001A), were used to infect RAW264.7 macrophages. All four isolates of *S *. *enterica* serovar Kentucky were able to associate, invade and replicate within the macrophages (Figs 5 and S3). The Kentucky-I isolate from broilers associated with macrophages better than the other three isolates. Importantly, on average, the two serovar Kentucky-II isolates were able to replicate within macrophages 8.6 times more than the Kentucky-I isolates.

### Distribution of virulence factors between serovar Kentucky lineages

**Fig. 6.**
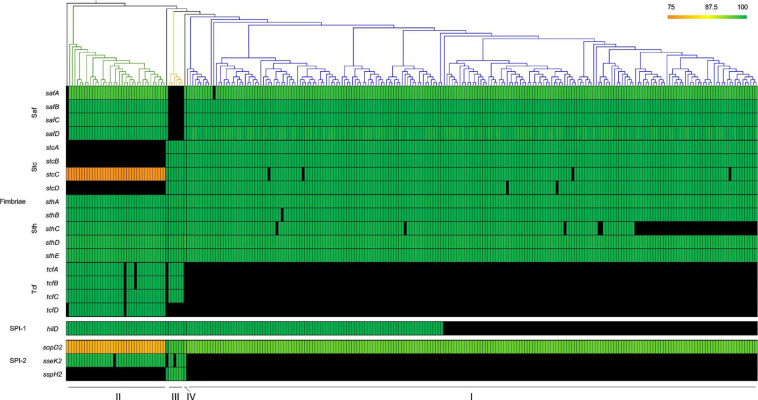
Unique distribution of fimbrial operons and virulence factors across serovar Kentucky lineages. Virulence factors with at least 75 % nucleotide identity against the VFDB are shown on a linear scale from 75 % (orange) to 100 % (green). Absent factors are shown in black. The colours used in the phylogenetic tree depict the three groups as in Fig. 1. Genomes from serovars Worthington and Agona, plus the outlier serovar Kentucky genome are also included.

The VFDB was used to screen putative virulence factors unique to *Salmonella *. The highly conserved *Salmonella* fimbrial operons *agf/csg*, *bcf* and *fim* were present in all serovar Kentucky genomes, as was the *lpf* operon (Fig. 6, Table S2). Similarly, the fimbrial operons *pef*, *sef* and *stg*, which are found in fewer serovars, were absent from all serovar Kentucky isolates. Presence or absence of other *Salmonella* fimbrial operons was largely conserved across all serovar Kentucky genomes with a few exceptions. For example, the *saf* operon was absent in Kentucky-III genomes, while *stc* was absent from Kentucky-II genomes. The *tcf* operon was entirely absent from Kentucky-I genomes and *tcfD* was missing in all Kentucky-III genomes. Additional virulence genes were unique to specific clades; *sseK2* and *sspH2* were both absent in Kentucky-I, and the latter was also absent in Kentucky-II. The *sopD2* gene, while found in all serovar Kentucky genomes, was best conserved in Kentucky-III genomes (96.8 % nucleotide identity) and least conserved in Kentucky-II genomes (79.8 % nucleotide identity). Finally, the majority of SPI-1 genes were present in all Kentucky isolates with the exception of the master regulatory gene, *hilD*, which was absent in approximately half of the Kentucky-I genomes, and also *spaS*, which was missing in a small subset of Kentucky-I genomes (Table S2).

## Discussion

It is becoming clear that a significant number of *Salmonella* serovars are polyphyletic, where they share the same O and H antigens but are genetically distinct and do not share a recent common ancestor. Genomic analyses have shown that polyphyly occurs frequently in *Salmonella* [11, 56, 57]. Because different serovars can have vastly different phenotypes, and because by definition polyphyletic serovars are evolutionarily separated by at least one other serovar, it is important to consider isolate characterization beyond serotyping to distinguish between different lineages of the same serovar [21]. Previous studies have shown that serovar Kentucky is polyphyletic and separated into two evolutionarily separate lineages, serovar Kentucky-I and serovar Kentucky-II [23, 24, 26]. The work presented here demonstrates that there are two additional lineages, which we called have serovar Kentucky-III and IV. Isolates belonging to different polyphyletic lineages have different CRISPR spacer content, with little or no overlap of spacers [20, 23, 58, 59]. Similarly, the allelic identifiers used to generate an MLST profile are also different across polyphyletic lineages [19, 60].

Data presented in this study show that serovar Kentucky continues to be the major serovar isolated from broilers compared to other domestic food animal sources [29]. In broilers, the primary serovar Kentucky isolates found belong to the Kentucky-I lineage, whereas serovar Kentucky-II are primarily cattle and human associated, which supports previous work [24, 61]. In an earlier study from our group that was focused on human and animal isolates from Pennsylvania (USA), all animal isolates were Kentucky-I, while human isolates were half Kentucky-I and half Kentucky-II. This led us to conclude that the human Kentucky-II cases were likely linked to international travel or consumption of imported food [27]. While international travel has been linked to human salmonellosis caused by serovar Kentucky-II isolates [37], the observation of isolates belonging to this lineage in domestic cattle by us here and by others suggests that some human cases could be caused by domestic food animal production [24]. In Europe, human Kentucky-II cases (ST198) are often linked to international travel [62]; however, studies have shown that this lineage is expanding significantly in poultry flocks in Europe [33, 63, 64]. More recently, ST198 isolates (Kentucky-II) have been found in cattle and also swine in Africa [65]. Furthermore, of the ten SNP clusters with multiple source types that belong to the Kentucky-II lineage, isolates from seven of them were only found in cattle and humans. However, for three of the shared SNP clusters between cattle and humans, the KCTs between genomes within the same cluster were different (e.g. for cluster PDS000032811.7, two genomes were included in this study and each had a different KCT; SRR12386238 was KCT55 and SRR10579773 was KCT70). Repetitive elements are masked from genome assemblies before Pathogen Detection adds them to the existing phylogenies via the SNP pipeline [66]. This can result in the absence of some genomic sequences containing repeats, such as prophages, transposons and CRISPRs. Therefore, while the genomes are very closely related, there are genetic differences that are not accounted for when a SNP cluster is designated. This explains why differences in the CRISPR arrays, resulting in different KCTs, occur in genomes within the same SNP cluster. Similarly, of the 37 SNP clusters with shared source types that belong to serovar Kentucky-I, 13 only contain isolates from broilers and humans but 8 of these had isolates in the same SNP cluster with different KCTs. This data collectively indicates that in the USA, cattle are the major animal reservoir for serovar Kentucky-II, while broilers are an animal reservoir for serovar Kentucky-I.

For serovar Kentucky-III, most isolates were found in humans, with genomes from one SNP cluster found in cattle and humans and both genomes also typed as ST314 and KCT71, suggesting that they are the same or very closely related strains. ST314 is an emerging Kentucky ST worldwide with reports of isolated strains internationally including Ireland, China and parts of South-East Asia [67–69]. Interestingly, a study in Ireland demonstrated that the majority of ST314 strains were isolated in non-human/non-clinical sources, mainly from animal/food sources. Our surveillance data reveals that ST314 was found in bovine and human cases in the USA, which was also reported in other studies [61, 69]. Continued surveillance is needed to determine whether ST314 will expand in the USA. There were two Kentucky-IV isolates (SRR5926911 and SRR10615607), and they were both isolated in humans and typed as ST696 and KCT74. According to EnteroBase, other serovar Kentucky ST696 isolates have been isolated from humans in the UK, Canada and Poland within the time frame of this study (2017–2021) and another study identified these from humans between 2008–2016 in the USA [61]. Moreover, all ST696 isolates on EnteroBase are attributed to humans, so it remains to be determined what or where the source of this particular ST is.

*Salmonella* spp. encode multiple different fimbrial operons, and different fimbriae have been associated with the ability to persist in mice and other animal hosts, and to invade epithelial cells [70–72]. Furthermore, SNP differences among different serovar Typhimurium isolates can drive adhesion within specific hosts [73]. There were 11 fimbrial operons that were present in all serovar Kentucky genomes, including *agf*/*csg*, *bcf*, *fim*, *std* and *ste*, and 5 that were absent in all genomes, *peg*, *pef*, *stg*, *sef* and *sta*. Differences among the three serovar Kentucky lineages include the *tcf* fimbrial operon that encodes the Typhi colonization factor, a chaperone-usher fimbria induced in nutrient-rich and microaerobic conditions used for host specificity in typhoidal serovars. In non-typhoidal *Salmonella* spp., the TcfA, TcfB and TcfC proteins show high conservation across multiple serovars; however, the adhesion portion of the operon, TcfD, shows sequence diversity, suggesting a degree of host tropism [74]. While host attachment and *Salmonella* colonization requires many factors, it is possible that the presence of this conserved operon in serovar Kentucky-II isolates enables them to adhere better to the intestine of cattle compared to chickens, and this may explain some of the host differences we observe in our surveillance data. Because fimbriae are required for host colonization, and because the *stc* fimbrial operon is present in Kentucky-I but absent in Kentucky-II, it is possible that this may explain the selective ability of these isolates to effectively colonize chickens compared to Kentucky-II. Although the *stc* operon is present in a number of *Salmonella* serovars, it is also present in serovars Enteritidis and Gallinarum, which both effectively colonize chickens [75]. This warrants further investigation and there are likely additional factors as this operon is also present in Kentucky-III genomes and isolates belonging to this new lineage were not found in poultry in our study.

Our cell assay showed that the two serovar Kentucky-II isolates were better able to proliferate in macrophages than their Kentucky-I counterparts. All *S. enterica* have SPI-2, which enables invasion and intracellular survival [76]. The absence of the gene encoding the secreted Type III secretion system (T3SS) effector protein SseK2 in Kentucky-I may explain why Kentucky-I is infrequently associate with human illness, as this effector contributes to bacterial translocation within host cells, early proliferation and biofilm formation in serovar Typhimurium [77, 78]. Conversely, while SopD2 is present in all three serovar Kentucky lineages, it is least conserved in Kentucky-II genomes, which was unexpected as deletion of this SPI-2 secreted effector in serovar Typhimurium limits replication in the same macrophage cell line as we used here [79]. A previous study comparing growth of serovars Typhimurium and Kentucky (lineage I) showed that serovar Typhimurium survived better in macrophages with a log difference in the total c.f.u. ml^−1^ of *Salmonella* recovered between the two serovars [80]. We did not observe such a large difference between Kentucky-I and -II isolates, suggesting that serovar Kentucky-II isolates are not able to replicate as well as serovar Typhimurium. That same study showed the differential absence of *sodCI*, *sopE* and *sseI* in serovar Kentucky compared to Typhimurium. These genes may influence relative virulence in Typhimurium [81, 82] as SodC1 is a metal superoxide dismutase that protects against host cell radicals [83], SopE is translocated upon initial host cell contact and creates a replicative niche [84], and SseI modulates dendritic cell activation to promote intracellular replication [85]. The *sodCI*, *sopE* and *sseI* genes were notably absent across all serovar Kentucky genomes we analysed. Further studies examining transcription of virulence factors during macrophage invasion and during replication are required to understand the interplay of these factors during serovar Kentucky pathogenesis. Although serovar Kentucky-I is missing a number of virulence factors, it can still cause illness in some individuals; therefore, the possibility of salmonellosis occurring in immunocompromised individuals where perhaps a whole suite of virulence factors is not required should be explored.

While the number of human salmonellosis cases caused by serovar Kentucky in the USA is low compared to other serovars, our surveillance data shows that isolates belonging to the Kentucky-II lineage more often cause human illness than those belonging to Kentucky-I. This increased pathogenic potential could be linked to the increased replication capacity we observed of Kentucky-II in macrophages. This also explains the higher association with human illness seen in Europe, where Kentucky-II is more prevalent than Kentucky-I. Pathogenic and host tropism differences among polyphyletic lineages of serovars have been previously observed, including for serovar Newport [12–14], which was the leading serovar associated with human illness in the USA in 2020 [86]. The work presented here highlights the importance of additional characterization beyond serotyping, especially for polyphyletic serovars of clinical importance such as serovar Enteritidis, Montevideo and Newport. For serovar Kentucky there is a need to detect isolates with a greater association with human illness [i.e. belonging to Kentucky-II (ST198) or -III (ST314)] and distinguishing these from less pathogenic isolates [i.e. Kentucky-I (ST152)]. This can be accomplished using molecular typing tools and can be easily derived from WGS data.

## Supplementary Data

Supplementary material 1

Supplementary material 2

Supplementary material 3
