# Supplementary material for: Genomic and phenotypic characterization of Salmonella enterica serovar Kentucky
Source: Microb Genom. 2023 Sep 26;9(9):001089. doi: 10.1099/mgen.0.001089 (PMC10569734; doi:10.1099/mgen.0.001089)
Supplement: Supplementary material 1 [file mgen-9-1089-s001.pdf]

**Supplemental Figure 1. Cladeogram showing *Salmonella* serovar Kentucky genomic structure.** kSNP was used to generate a SNP matrix for 409 serovar Kentucky genomes plus two outgroup genomes (serovar Worthington ATCC9607 and Agona ATCC51957). These genomes represent multiple animal sources from each SNP cluster (260 genomes) as well as isolates with no designated SNP cluster (147 genomes). The phylogenetic tree was generated using RAxML and visualized using iTOL; bootstrap values greater than or equal to 70 are shown. Serovar Kentucky-I genomes are indicted by blue branches, Kentucky-II by green branches, and the new lineage, Kentucky-III by orange branches. Shapes in the inner most ring represent the source, as indicated. White shapes represent a SNP cluster that only contained isolates from that source. Coloured shapes represent multi-source SNP clusters. The next ring shows the SRR number associated with each genome. The third ring represents the Achtman MLST and each ST is colour coded as indicated.

**Supplemental Figure 2. *Salmonella* serovar Kentucky CRISPR arrays separate three main serovar Kentucky lineages**. Spacer composition of both CRISPR arrays, CRISPR1 and CRISPR2, is shown. Each unique spacer is represented by a uniquely coloured box and the shape inside each box denotes the length of the spacer. The direct repeats are omitted for clarity. The spacers are aligned from 5' to 3' with gaps representing the absence of a spacer. Each allele represents a unique composition of spacers in the CRISPR array. Allelic identifiers for CRISPR1 and CRISPR2 are combined to generate a unique Kentucky CRISPR Type (KCT).

**Supplemental Figure 3. *Salmonella* serovar Kentucky-II isolates demonstrate higher replication in macrophages in two additional replicates (A and B).** Four *Salmonella* serovar Kentucky isolates were used to infect RAW264.7 murine macrophage cells and Salmonella attachment, invasion, and replication were measured. Error bars represent standard error of three technical triplicate experiments. The Tukey-Kramer statistical test was used and significance represented by lowercase letters, with identical letters determining significant similarity between each stage (association, invasion, replication).

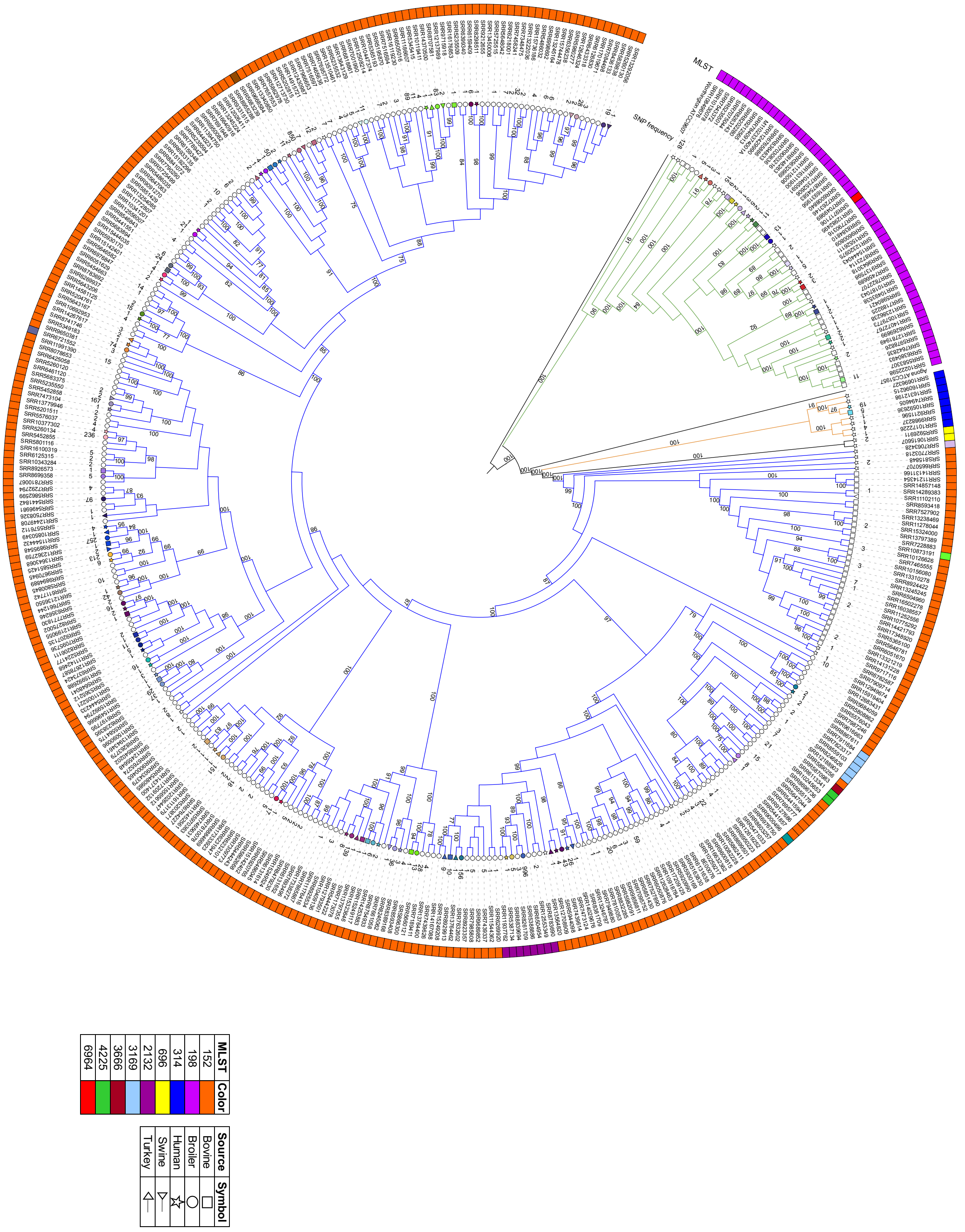
MLST
SNP frequency
MLST
Color
152
198
314
696
2132
3169
3666
4225
6964
Source
Symbol
Bovine
Broiler
Human
Swine
Turkey

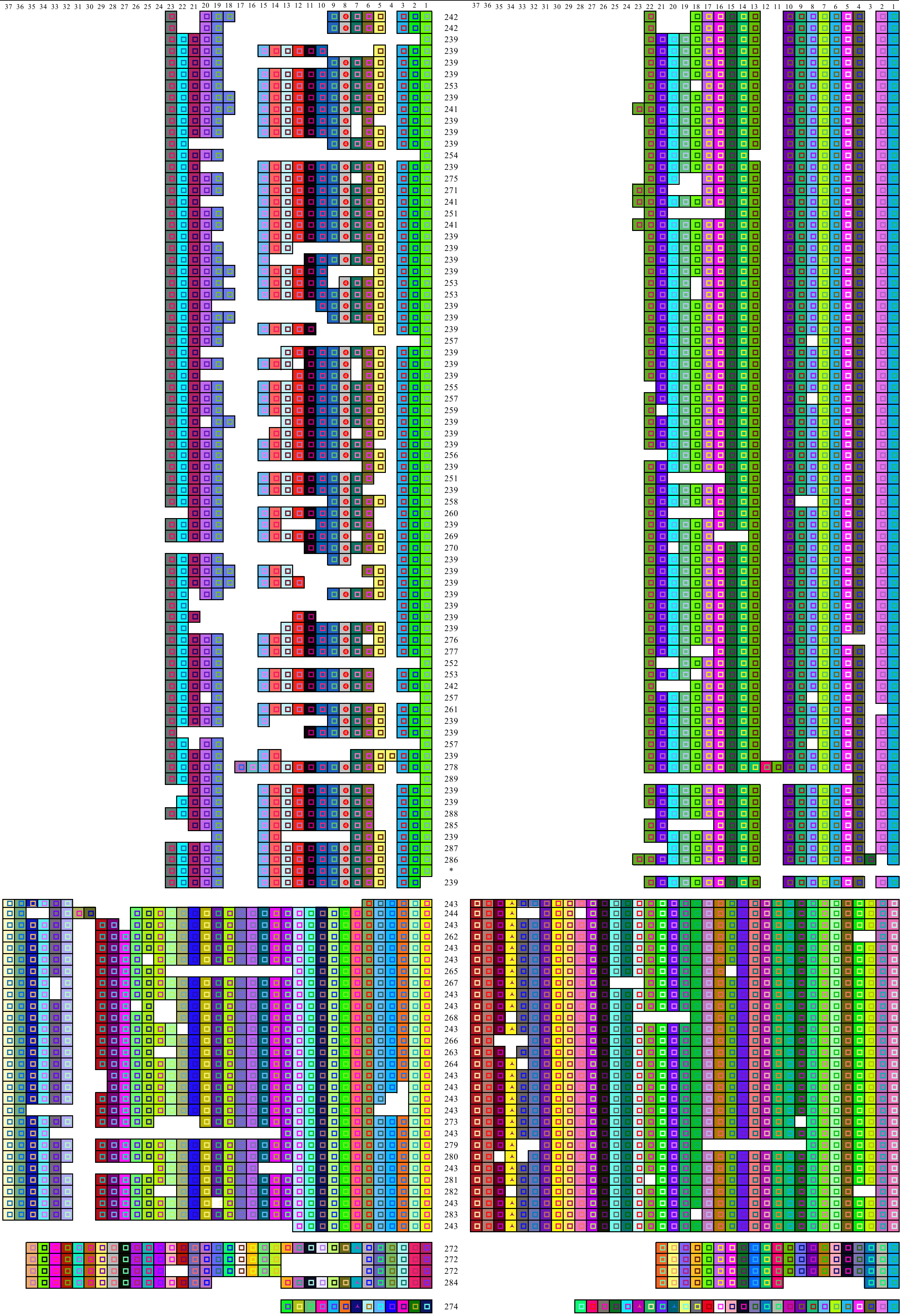


| Group | Frequency | Kentucky CRISPR Type (KCT) | CRISPR1 Allele | CRISPR2 Allele |
|---|---|---|---|---|
| Kentucky-I | 2 | 1 | 210 | 242 |
| | 2 | 2 | 229 | 242 |
| | 19 | 3 | 206 | 239 |
| | 3 | 4 | 230 | 239 |
| | 12 | 5 | 231 | 239 |
| | 116 | 6 | 224 | 239 |
| | 8 | 7 | 224 | 253 |
| | 31 | 8 | 209 | 239 |
| | 13 | 9 | 209 | 241 |
| | 1 | 10 | 233 | 239 |
| | 40 | 11 | 205 | 239 |
| | 1 | 12 | 232 | 239 |
| | 1 | 13 | 206 | 254 |
| | 4 | 14 | 222 | 239 |
| | 1 | 15 | 205 | 275 |
| | 1 | 16 | 224 | 271 |
| | 2 | 17 | 222 | 241 |
| | 1 | 18 | 224 | 251 |
| | 2 | 19 | 224 | 241 |
| | 2 | 20 | 235 | 239 |
| | 1 | 21 | 236 | 239 |
| | 1 | 22 | 237 | 239 |
| | 2 | 23 | 239 | 239 |
| | 1 | 24 | 240 | 253 |
| | 1 | 25 | 209 | 253 |
| | 1 | 26 | 241 | 239 |
| | 3 | 27 | 227 | 239 |
| | 1 | 28 | 243 | 239 |
| | 5 | 29 | 206 | 257 |
| | 1 | 30 | 234 | 239 |
| | 1 | 31 | 244 | 239 |
| | 1 | 32 | 245 | 239 |
| | 1 | 33 | 224 | 255 |
| | 1 | 34 | 224 | 257 |
| | 1 | 35 | 224 | 259 |
| | 1 | 36 | 246 | 239 |
| | 2 | 37 | 247 | 239 |
| | 14 | 38 | 204 | 239 |
| | 1 | 39 | 224 | 256 |
| | 1 | 40 | 248 | 239 |
| | 2 | 41 | 204 | 251 |
| | 1 | 42 | 249 | 239 |
| | 1 | 43 | 231 | 258 |
| | 1 | 44 | 250 | 260 |
| | 2 | 45 | 242 | 239 |
| | 1 | 46 | 205 | 269 |
| | 1 | 47 | 238 | 270 |
| | 1 | 48 | 268 | 239 |
| | 1 | 49 | 263 | 239 |
| | 1 | 50 | 264 | 239 |
| | 1 | 75 | 270 | 239 |
| | 1 | 76 | 271 | 239 |
| | 1 | 77 | 272 | 239 |
| | 1 | 78 | 273 | 239 |
| | 1 | 79 | 274 | 276 |
| | 1 | 80 | 224 | 277 |
| | 5 | 81 | 206 | 252 |
| | 1 | 82 | 204 | 253 |
| | 1 | 83 | 204 | 242 |
| | 2 | 84 | 275 | 257 |
| | 1 | 85 | 224 | 261 |
| | 1 | 86 | 276 | 239 |
| | 1 | 87 | 277 | 239 |
| | 1 | 88 | 278 | 257 |
| | 1 | 89 | 279 | 239 |
| | 2 | 90 | 280 | 278 |
| | 1 | 107 | 206 | 289 |
| | 1 | 100 | 284 | 239 |
| | 1 | 101 | 288 | 239 |
| | 1 | 102 | 204 | 288 |
| | 1 | 103 | 286 | 285 |
| | 1 | 104 | 287 | 239 |
| | 1 | 105 | 224 | 287 |
| | 1 | 106 | 224 | 286 |
| | 1 | - | 224 | * |
| | | 109 | 291 | 239 |
| Kentucky-II | 1 | 51 | 258 | 243 |
| | 1 | 52 | 212 | 244 |
| | 2 | 53 | 254 | 243 |
| | 1 | 54 | 216 | 262 |
| | 18 | 55 | 216 | 243 |
| | 2 | 56 | 253 | 243 |
| | 2 | 57 | 256 | 265 |
| | 1 | 58 | 213 | 267 |
| | 2 | 59 | 213 | 243 |
| | 2 | 60 | 257 | 243 |
| | 1 | 61 | 255 | 268 |
| | 1 | 62 | 252 | 243 |
| | 1 | 63 | 216 | 266 |
| | 2 | 64 | 255 | 263 |
| | 2 | 65 | 216 | 264 |
| | 1 | 66 | 259 | 243 |
| | 1 | 67 | 260 | 243 |
| | 1 | 68 | 261 | 243 |
| | 1 | 69 | 262 | 243 |
| | 1 | 70 | 266 | 273 |
| | 1 | 91 | 282 | 243 |
| | 1 | 92 | 216 | 279 |
| | 1 | 93 | 216 | 280 |
| | 1 | 94 | 283 | 243 |
| | 1 | 95 | 216 | 281 |
| | 1 | 96 | 255 | 282 |
| | 1 | 97 | 281 | 243 |
| | 1 | 98 | 216 | 283 |
| | 1 | 108 | 289 | 243 |
| Kentucky-III | 3 | 71 | 251 | 272 |
| | 3 | 72 | 265 | 272 |
| | 1 | 73 | 267 | 272 |
| | 1 | 99 | 285 | 284 |
| IV | 2 | 74 | 269 | 274 |

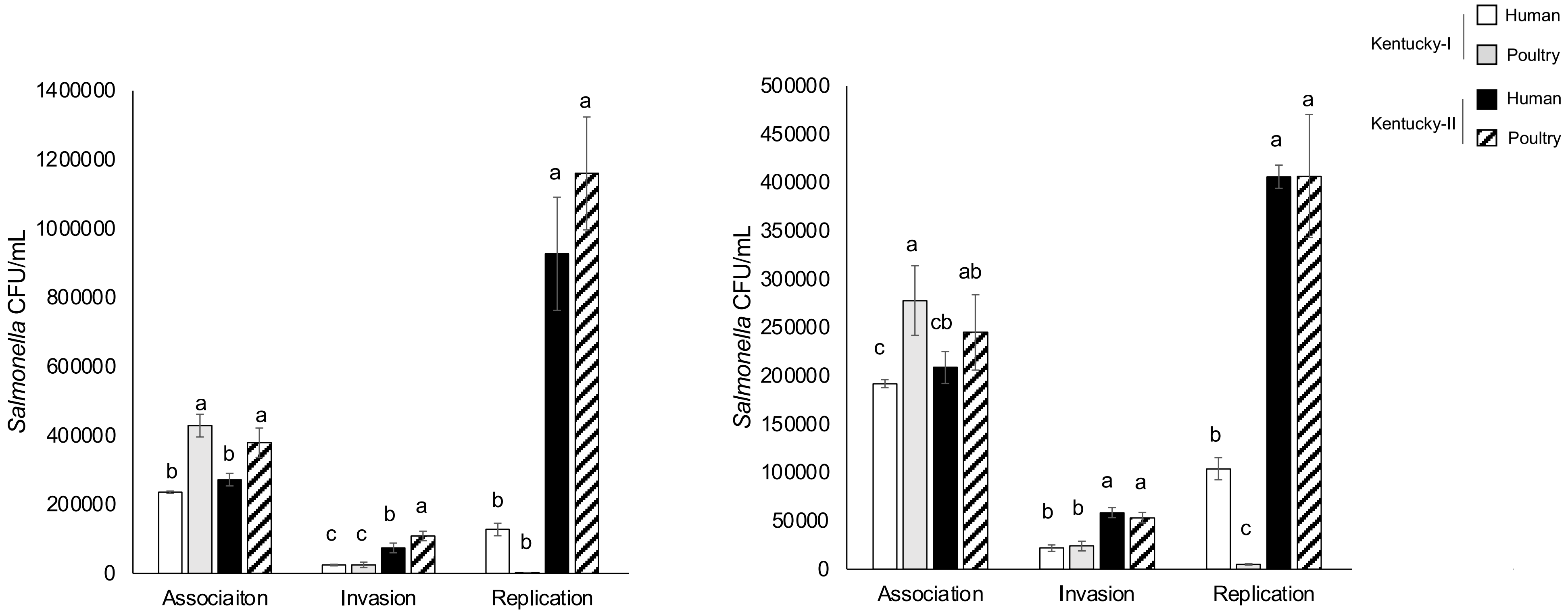
Salmonella CFU/mL
1400000
1200000
1000000
800000
600000
400000
200000
0
Associaiton
Invasion
Replication
Salmonella CFU/mL
500000
450000
400000
350000
300000
250000
200000
150000
100000
50000
0
Association
Invasion
Replication
Kentucky-I
Human
Poultry
Kentucky-II
Human
Poultry
